# A Biological Circuit Involving Mef2c, Mef2d, and Hdac9 Controls the Immunosuppressive Functions of CD4+Foxp3+ T-Regulatory Cells

**DOI:** 10.3389/fimmu.2021.703632

**Published:** 2021-07-05

**Authors:** Eros Di Giorgio, Liqing Wang, Yan Xiong, Lanette M. Christensen, Tatiana Akimova, Rongxiang Han, Arabinda Samanta, Matteo Trevisanut, Claudio Brancolini, Ulf H. Beier, Wayne W. Hancock

**Affiliations:** ^1^ Division of Transplant Immunology, Department of Pathology and Laboratory Medicine, Children’s Hospital of Philadelphia and Perelman School of Medicine, University of Pennsylvania, Philadelphia, PA, United States; ^2^ Department of Medicine, University of Udine, Udine, Italy; ^3^ Institute of Hepatobiliary Diseases of Wuhan University, Transplant Centre of Wuhan University, Zhongnan Hospital of Wuhan University, Wuhan University, Wuhan, China; ^4^ Division of Nephrology, Department of Pediatrics, Children’s Hospital of Philadelphia and Perelman School of Medicine, University of Pennsylvania, Philadelphia, PA, United States

**Keywords:** Mef2c, Mef2d, IL-10, Icos, anti-cancer immunity, Treg

## Abstract

The Mads/Mef2 (Mef2a/b/c/d) family of transcription factors (TFs) regulates differentiation of muscle cells, neurons and hematopoietic cells. By functioning in physiological feedback loops, Mef2 TFs promote the transcription of their repressor, Hdac9, thereby providing temporal control of Mef2-driven differentiation. Disruption of this feedback is associated with the development of various pathologic states, including cancer. Beside their direct involvement in oncogenesis, Mef2 TFs indirectly control tumor progression by regulating antitumor immunity. We recently reported that in CD4+CD25+Foxp3+ T-regulatory (Treg) cells, Mef2d is required for the acquisition of an effector Treg (eTreg) phenotype and for the activation of an epigenetic program that suppresses the anti-tumor immune responses of conventional T and B cells. We now report that as with Mef2d, the deletion of Mef2c in Tregs switches off the expression of *Il10* and *Icos* and leads to enhanced antitumor immunity in syngeneic models of lung cancer. Mechanistically, Mef2c does not directly bind the regulatory elements of *Icos* and *Il10*, but its loss-of-function in Tregs induces the expression of the transcriptional repressor, Hdac9. As a consequence, Mef2d, the more abundant member of the Mef2 family, is converted by Hdac9 into a transcriptional repressor on these loci. This leads to the impairment of Treg suppressive properties *in vivo* and to enhanced anti-cancer immunity. These data further highlight the central role played by the Mef2/Hdac9 axis in the regulation of CD4+Foxp3+ Treg function and adds a new level of complexity to the analysis and study of Treg biology.

## Introduction

The role played by Treg cells in restraining anti-tumor immunity (1) and in limiting transplant rejection (2) is well established and has made the study of Treg differentiation, stability, subpopulations and homeostasis a major focus of immunology (3). Foxp3 is the master transcription factor (TF) involved in sustaining Treg suppressive identity and mutations leading to Foxp3 loss-of-function are associated clinically with the severe autoimmunity that presents as IPEX syndrome (Immune dysregulation, Polyendocrinopathy, Enteropathy, X-linked) (4–6). However, Foxp3 has been demonstrated to be necessary, but not sufficient, for triggering and maintaining the Treg phenotype (7). The exceptional interest in Treg biology has made Foxp3 one of the most studied TFs so far (8). Foxp3 transcriptional activities are finely modulated through four main mechanisms: i) the transcriptional and post-translational regulation of its expression (9); ii) the heterogeneity of Foxp3 partners involved in sustaining (9), but also positively or negatively modulating its transcriptional functions (10); iii) the large number of epigenetic regulators that act as pioneering factors (11, 12) or plastically modulate Foxp3 activities (13, 14); and iv) the protein-protein complexes and transcription factors that antagonistically or agonistically integrate Foxp3 responses (15–17).

We recently identified Mef2d as a TF that supports and integrates the transcriptional responses of Foxp3, allowing Tregs to acquire the phenotype of effector Tregs (eTregs) (18). Similarly to Foxp3, the four Mef2 paralogues (Mef2a, b, c, d) assemble into multi-protein complexes which modulate the resulting transcriptional responses to control various and complex differentiative and adaptive programs (19, 20). The dynamic nature of Mef2 protein complexes has been little investigated in T effector cells and is completely unexplored in Treg cell. Here, by exploring the roles of Mef2c in CD4+ Foxp3+ Treg cells, we discovered the existence of a feed-back circuit involving Mef2c, Mef2d and Hdac9. Interference in this circuit decreases the immunosuppressive properties of Foxp3+ Tregs *in vivo*. Our results stress the need to deepen the study of this signaling pathway so as to more fully understand the transcriptional dynamics of Treg cells.

## Materials and Methods

### Mice

BALB/c and C57BL/6 mice were purchased from The Jackson Laboratory. Foxp3*^YFP-cre^* mice (21) and Mef2c*^flox/flox^* mice (22) were previously described and backcrossed on the C57BL/6 background at least 8 times. Mice with conditional deletion of Mef2c within their Foxp3+ Treg cells (Foxp3*^YFP-cre^*Mef2c*^flox/flox^*) are hereafter listed as Mef2c-/- mice. Foxp3^YFP-cre^ mice were used as wild-type controls.

### Co-Immunoprecipitation and Western Blotting

Teff and Treg cells were lysed with hypotonic buffer (20 mM Tris-HCl, pH 7.5; 2 mM EDTA; 10 mM KCl; 1% Triton X-100), supplemented with protease and phosphatase inhibitors; 1 μg of antibody (Ab) was used for immunoprecipitation, and Protein-G agarose (Invitrogen, #15920-010) was used to collect Ab-antigen complexes. Cell lysates were separated by SDS-PAGE, transferred to nitrocellulose membranes and immunoblotted with the following Abs: Mef2d (Becton-Dickinson, #610774), Foxp3 (Invitrogen, #700914), β-actin (Cell Signaling Technology, #3700), and Hdac9 (23). Secondary HRP-conjugated Abs to mouse (#7076), rat (#7077) and rabbit (#7074) IgG were purchased from Cell Signaling Technology. Unconjugated CD3 (clone 145-2C11, #553057) and CD28 (clone 37.51, #553294) mAbs used for cell activation were purchased from Becton-Dickinson.

### Flow Cytometry

Single-cell suspensions from secondary lymphoid tissues or tumors were prepared as previously described (15) and stained with fluorochrome-conjugated mAbs directed against CD4 (Pacific blue, Invitrogen, #MHCD0428), CD8 (Super Bright 645, eBioscience, clone 53-6.7, #64-0081-82), Foxp3 (eFluor 450, eBioscience, clone FJK-16s, #48-5773-82 and PE-Cy5 #15-5773-82), CD62L (PE-Cy7, clone MEL-14, #25-0621-82), IFN-γ (APC, clone XMG1.2, #554413; PE # 554412), CD44 (PE-Cy5, eBioscience, clone IM7, #15-0441-83), Ctla4 (APC, #17-1522-82), Icos (PE, # 12-9949-81), CD25 (APC, eBioscience, clone PC61.5, #17-0251-82), and CD8a (FITC, #53-6.7), and acquired on a Cytoflex (Beckman Coulter) flow cytometer.

### Treg Suppression Assays

5×10^4^ CD4+CD25- conventional T cells and CD4+CD25+Tregs from Foxp3^YFP-Cre^ and Mef2c-/-mice were isolated using CD4+CD25+Treg isolation kits (Miltenyi Biotec, #130-091-041) and seeded into 96-well plates. Equal numbers of CFSE-labeled CD4+CD25- T cells and γ-irradiated antigen-presenting cells (CD90.2^–^, Miltenyi Biotec, #130-049-101), plus CD3 mAb (1 μg/ml), were cultured for 72 h with different concentrations of Tregs. After 72 h, proliferation of conventional T cells was determined by flow cytometry and analysis of CFSE dilution.

### Cardiac Transplantation

We undertook heterotopic cardiac allografting using BALB/c mice as donors and WT or Mef2c-/- C57BL/6 mice as recipients. On the day of engraftment, recipients were treated i.v. with CD154 mAb (Bio X Cell, clone MR-1, #BE0017-1, 200 µg) plus 5×10^6^ donor splenocytes (DST) (24). Allograft survival was monitored by palpation of ventricular contractions and subsequently confirmed by histological evaluation.

### ChIP Assays

Each ChIP was performed using 3×10^6^ Tregs. After 15’ of fixation with 1% formaldehyde and 30 cycles of sonication (30 sec ON and 30 sec OFF, Bioruptor, Diagenode), the resulting chromatin was immunoprecipitated using Abs against H3K27ac (2µg, Abcam #ab4729), Mef2d (5 µg, BD, # 610774), Mef2c (5 µg) (25), and Hdac9 (5 µg) (23). The immunoprecipitated DNA was purified and analyzed by qPCR (SYBR green, KAPA).

### RNA-Seq and Real-Time qPCR

RNeasy kits (QIAGEN) were used to isolate mRNA. mRNA with a RIN>7 were used to prepare libraries and perform RNA-sequencing by Novogene (Sacramento, CA) on the Illumina Platform PE150. The edgeR package was used to identify the differentially expressed genes (p-value <0.05 and fold change >1.3). GSEA (26) was performed to interrogate datasets with defined genesets, as described (27). The expression levels of individual genes were verified by qPCR. For this purpose, RNA was reverse transcribed to cDNA (Applied Biosystems) and Taqman primers and probe sets were used to perform RT-qPCR. Data were normalized to endogenous 18s and relative expression was determined by the formula 2^–ΔCT^.

### Cell Lines and Tumor Models

The murine lung adenocarcinoma cell line, TC1 (28), was provided by Dr. Yvonne Patterson (UPenn, Philadelphia, PA). Lung cancer cells were grown in RPMI supplemented with 10% fetal bovine serum (FBS), 2 mM glutamine, and 5 μg/ml penicillin & streptomycin. For lung tumor cells, each mouse was injected s.c. with 1.2×10^6^ TC1 tumor cells. Tumor volume was determined by the formula: ((short diameter)^2^ × long diameter)/2.

### Statistical analysis

Data were analyzed using GraphPad Prism 8.0 and Excel. Data are presented as mean ± standard error. Statistical comparisons between two groups were done with a 2-tailed Student’s t test. Comparisons between multiple samples were performed by using a 1-way ANOVA test with corresponding Tukey’s multiple comparison test. Graft survival was evaluated with Kaplan-Meier followed by log-rank test. We marked with *p<0.05, **p<0.01, ***p<0.005.

### Study Approval

Animal studies were approved by the Institutional Animal Care and Use Committee of the Children’s Hospital of Philadelphia (protocols 17-001047 and 19-000561).

### Primers

The following primers were used for ChIP-qPCR analysis: Itk FW: GTGCGACTGAAGGAGAGGAG, Itk RV: CATCAGAGGAGGGAGCTCAG, Hdac9 FW: CTCCAGAGGGTGTCCTCCTA, Hdac9 RV: GGCTTTGGTGGGGTATTTTT, Icos 1 FW: CCTCAGTCAGAAGGGTCGTC, Icos 1 RV: CAGAAATTCCTGGTCATGTTTT, Icos 2 FW: AGTCTGCCATAGGGTTGGTG, Icos 2 RV: TCAGTCATTTTCTCCCCCTTT, Il10 FW: TCTTTAGCGCTTACAATGCAAA, Il10 RV: CTGTTCTTGGTCCCCCTTTT.

## Results

### Mef2c Deletion in Tregs Only Partially Represses Their Suppressive Properties *In Vitro*

**Figure 1:**
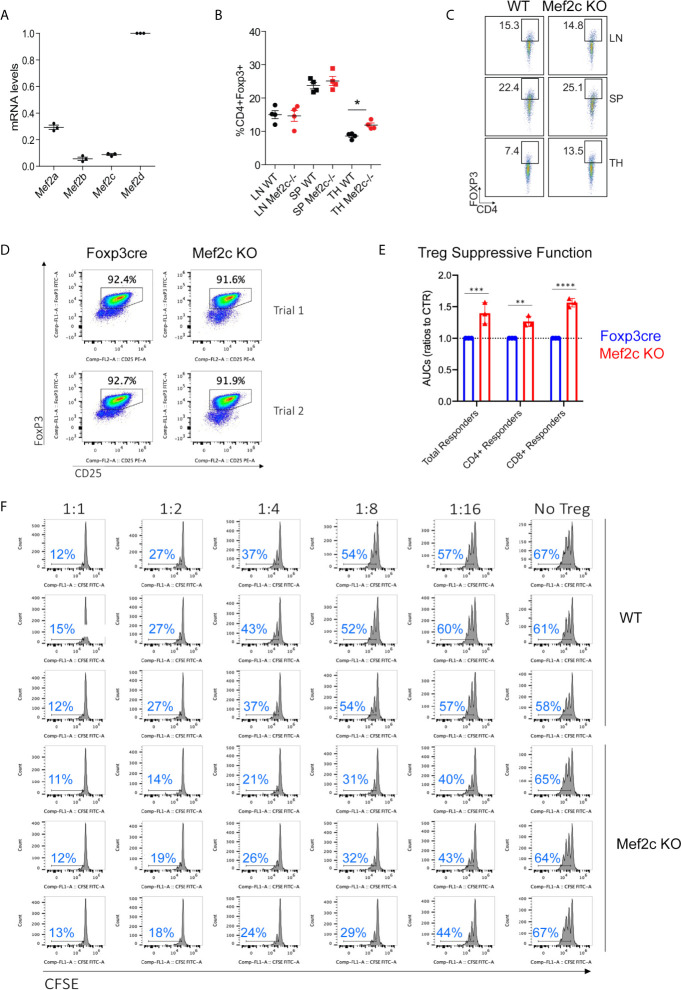
Mef2c deletion in Tregs increases their suppressive properties *in vitro*. **(A)** mRNA absolute levels of the indicated Mef2 paralogues in Tregs, expressed as RPKM levels relative to Mef2d levels. **(B, C)** Analysis of CD4+Foxp3+ in lymph nodes (LN), spleens (SP) and thymii (TH) tissues from Mef2c-/- or WT mice (n = 4, *p < 0.05, t-test). **(D)** Purity of CD4+CD25+Foxp3+ Tregs used for Treg assays (representative of 3 mice/group) and performed twice (Trial 1 and Trial 2). **(E)** AUC data from Treg suppression assays performed twice and in triplicate (**p < 0.01, ***p < 0.005, ***p < 0.001). **(F)** Examples of Treg suppression assays performed in triplicate and using all T cells as proliferating cells (separate CD4 and CD8 T cells responses are shown in **Supplementary Figure 1**); the proportion of proliferating T cells within each panel is shown in blue.

As noted in the literature, the various paralogues of MEF2 can play redundant (29, 30) or differentiative and adaptive roles (31, 32). Despite its low expression in murine Tregs (**Figure 1A**), we deleted Mef2c expression in Foxp3+ Treg cells by crossing Mef2c^fl/fl^ mice and Foxp3^YFP/Cre^ mice so as to investigate the existence of alternative, supplementary or redundant transcriptional programs with respect to those controlled by Mef2d, its more abundant paralogue. Mef2c^fl/fl^ Foxp3^YFP/Cre^ (hereafter Mef2c-/*-*) mice were born at expected Mendelian ratios and were characterized by normal numbers of Tregs in secondary lymphoid tissues, though CD4+Foxp3+ Tregs were increased in the thymus of Mef2c-/- mice (**Figures 1B, C**). Whether this was due to increased generation, stability or re-circulation to the thymus is presently unknown, but was previously also observed in Mef2d^fl/fl^Foxp3^YFP/Cre^ mice (18). The *in vitro* suppressive functions of Mef2c-/- Tregs were increased, as shown by cell-sorting (**Figure 1D**) and testing of the ability of purified Tregs to inhibit the proliferation of conventional T cells. Area-under-curve data are shown in **Figure 1E** and a triplicate assay, representative of 2 such assays, is shown in **Figure 1F**. Assessment of suppressive activity again CD4 and CD8 T cell subsets is shown in **Supplementary Figure 1**. Hence, while Mef2d deletion in Tregs led to decreased suppressive function *in vitro* (18), Mef2c deletion had an opposite effect.

### General Mef2 Signatures but Also Treg-Specific Signatures Are Altered in Mef2c-/- Tregs

**Figure 2:**
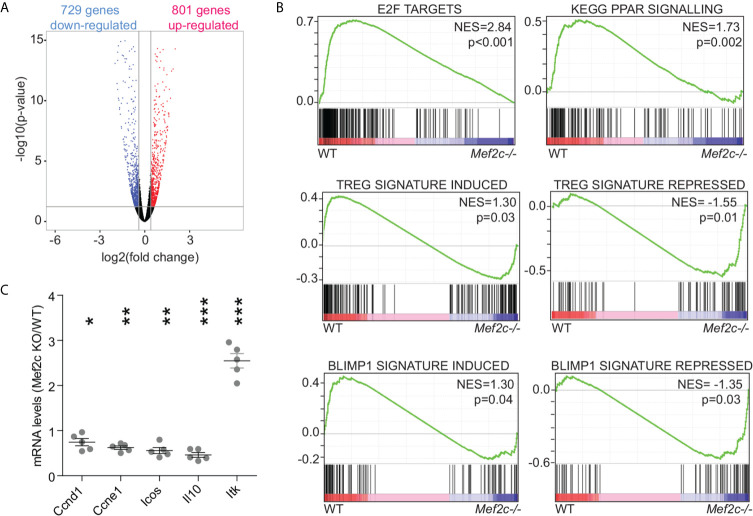
General Mef2 signatures but also Treg-specific signatures are altered in Mef2c-/- Tregs. **(A)** Volcano plot illustrating the statistical significance and the fold change for genes differentially expressed in Mef2c-/- Foxp3+ Tregs in respect to WT Treg cells. **(B)** GSEA plots obtained by using the indicated gene-sets and as dataset the transcriptome of WT or Mef2c-/- Tregs. **(C)** mRNA expression levels of the indicated genes, expressed as a ratio between Mef2c-/- and WT Tregs (t-test between the two groups for each gene, *p < 0.05, **p<0.01, ***p<0.005).

Despite the low expression levels of Mef2c in murine Tregs, the impact of Mef2c deletion on the Treg transcriptome was very similar to what we observed upon Mef2d deletion (18). That is, Mef2d deletion caused the up-regulation of 795 transcripts and the repression of 700 genes (18), while Mef2c deletion caused the up-regulation of 801 genes and the repression of 729 genes (>1.3-fold, p<0.05) (**Figure 2A**). Gene set enrichment analysis (GSEA) showed that in Tregs, Mef2c controls general transcriptional responses previously associated with Mef2 TFs in different cell types, like E2F and peroxisome proliferator-activated receptor-γ agonists/retinoic acid signaling (**Figure 2B**). Alongside the perturbation of these signaling pathways, we found that Mef2c controls the expression of more specific signatures related to the activation of Tregs and the execution of their immunosuppressive properties (**Figure 2B**). This analysis suggests that Mef2c is required to sustain the activation of Tregs and the transcriptional program activated by Blimp1 (**Figure 2B**). We validated the RNA-seq data by means of qRT-PCR on a selected pool of transcripts found to be dysregulated after Mef2c depletion; in each case there was agreement with the high-throughput data (**Figure 2C**).

### Mef2c Depletion Switches on Hdac9 Transcription and Triggers a Feedback Response That Limits the Transcriptional Activity of Mef2d

**Figure 3:**
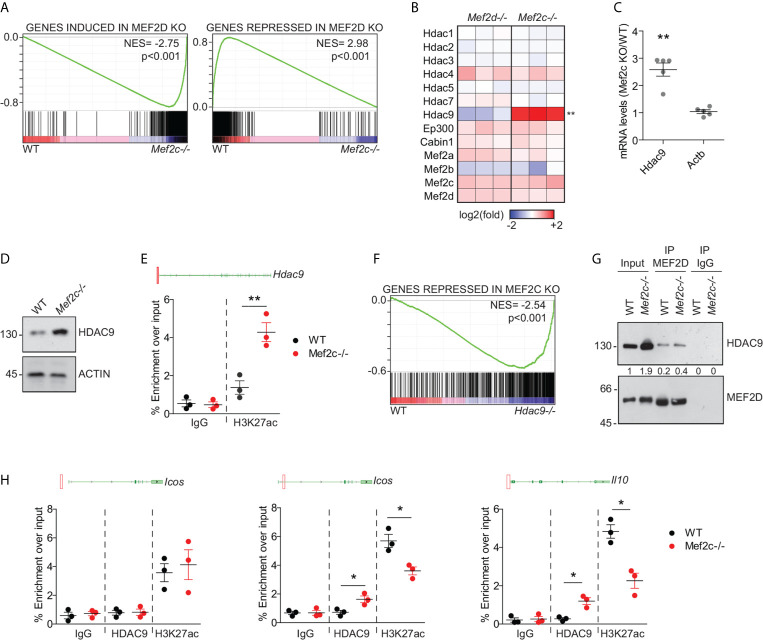
Mef2c depletion switches on Hdac9 transcription and triggers a feedback response that limits the transcriptional activity of Mef2d. **(A)** GSEA plots obtained by using the transcripts significantly induced (left) or repressed (right) in Mef2d-/- Tregs (18) as gene-sets and the transcriptome of WT or Mef2c-/- Tregs as dataset. **(B)** Heat-map representing the z-scores of the indicated transcripts in Mef2d-/- and Mef2c-/- Tregs in respect to WT Tregs. **(C)** mRNA expression levels of the indicated genes, expressed as a ratio between Mef2c-/- and WT Tregs (t-test between the two groups for each gene, n = 5, **p < 0.01). **(D)** Immunoblot analysis of HDAC9 in WT and Mef2c-/- Tregs, as indicated. Actin was used as loading control. **(E)** Histogram representing the qPCR results obtained in freshly isolated WT and Mef2c-/- Tregs after the ChIP with H3K27ac or IgG antibodies. Above the histogram we included the representation of the genomic locus of Hdac9. The red square indicates the position of the amplified region in respect to the leading TSS (n = 3, **p < 0.01). **(F)** GSEA plot obtained by using the transcripts significantly repressed in Mef2c-/- Tregs as gene-set and the transcriptome of WT or Hdac9-/- Tregs (33) as dataset. **(G)** Lysates from freshly isolated WT or Mef2c-/- Tregs were pulled down with anti-Mef2or IgG Ab (1μg). 1/50 total lysates have been included and marked as input. **(H)** Histogram representing the qPCR results obtained in freshly isolated WT and Mef2c-/- Tregs after the ChIP with the indicated antibodies. For each gene analyzed, we included the illustration of the genomic locus in which the red squares point to the position of the amplified region in respect to the leading TSS (n = 3, *p < 0.05).

GSEA analysis showed that many aspects of the transcriptional perturbation obtained after the depletion of Mef2c in Tregs are superimposable to that obtained by depleting Mef2d (**Figure 3A**). We hypothesized that this could be due to the re-organization of Mef2d transcriptional complexes achieved as a consequence of Mef2c KO. Though suggested many times in the literature (23, 27, 31, 34–37), the stoichiometry as well as the dynamic composition of MEF2 heterodimeric complexes in living cells have never been investigated in detail. We approached this issue by looking at the perturbations of mRNA abundance of the privileged partners of MEF2 TFs observed after Mef2c or Mef2d depletion (**Figure 3B**). Among the partners analyzed, only Hdac9 displayed differences between the two KOs, being induced after Mef2c deletion (**Figure 3B**). We confirmed this upregulation in Mef2c-/- Tregs both at the RNA (**Figure 3C**) and protein (**Figure 3D**) levels, and that upregulation was likely to be due to increased transcription as a consequence of the establishment of a proficient chromatin environment at the promoter level (**Figure 3E**).

Increased levels of Hdac9 can flip the balance of protein complexes assembled on Mef2d, thus altering its transcriptional effects (23, 38, 39). Indeed, most of the transcripts repressed in Mef2c-/- Tregs (**Figure 3F**) were upregulated in Hdac9-/- Tregs (33, 40). Levels of Mef2d engaged in protein complexes with Hdac9 in Mef2c-/- Tregs were almost double that of WT Tregs (**Figure 3G**). In Mef2c-/- Tregs, this resulted in the establishment of a repressive chromatin at the level of the Mef2d-bound (18) regulative elements of Icos and IL-10 (**Figure 3H**). The co-occurrence of Hdac9 binding suggests, but does not directly prove, its involvement in decreasing the acetylation of H3K27 on these loci (**Figure 3H**). These data suggest that the release of Hdac9 transcription achieved after Mef2c KO could be involved in the establishment of repressive complexes on Mef2d, thus leading to the perturbation of eTreg properties, similarly to what we reported for Mef2d-/- Tregs (18).

### Mef2c Deletion Dampens Treg Function *In Vivo*

We used two animal models to assess the effects of Mef2c deletion on Treg suppressive functions *in vivo*.

**Figure 4:**
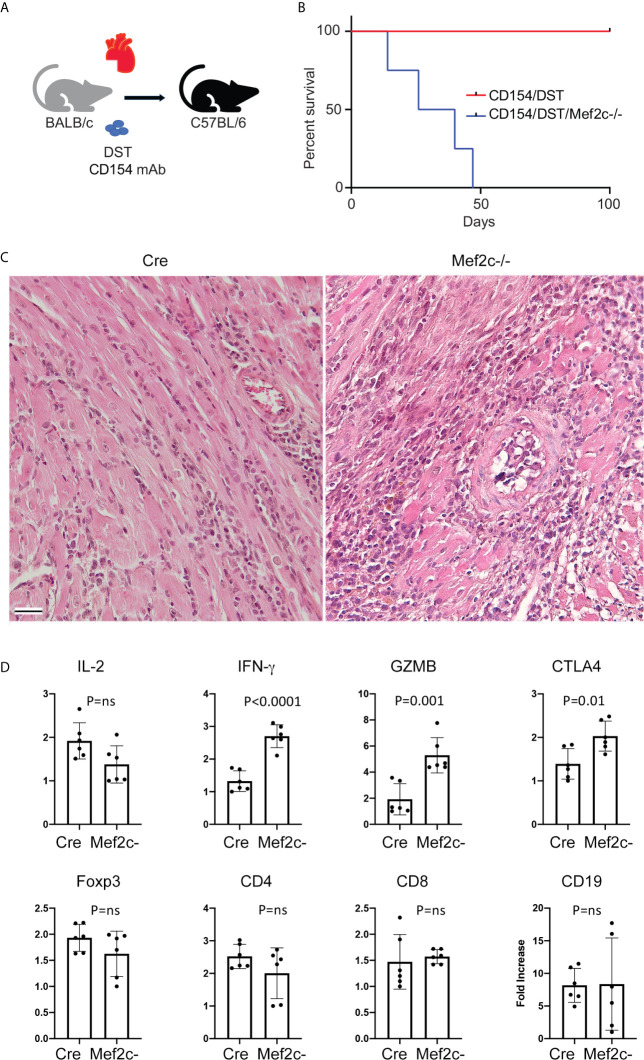
Mef2c deletion dampens Treg function *in vivo*. **(A)** Schematic illustrating cardiac allografting procedure. **(B)** Differently from WT recipients, Mef2c-/- mice acutely rejected cardiac allografts (BALB/c->C57BL/6) despite costimulation blockade with CD154 mAb/DST; n = 5/group (Tukey’s multiple comparison test, p < 0.01). The cardiac allografting was repeated twice with similar results. **(C)** Histology of transplanted hearts show focal interstitial infiltrates in WT recipients (Cre) and dense mononuclear infiltrates with multifocal myocyte necrosis and vascular injury in conditionally deleted Mef2c-/- recipients (bar = 100 µM). **(D)** qPCR results of the expression of the indicated genes in samples collected 21 days after cardiac allografting in WT (Cre) and Mef2c–/– mice (n = 6/group). ns, not significant.

First, we performed cardiac allografting using BALB/c donors and WT or Mef2c-/- C57BL/6 recipients in the presence of co-stimulation blockade with CD154 mAb/DST (**Figure 4A**) (24). While co-stimulation blockade induced long-term allograft survival (>100 d) in WT recipients, acute rejection was observed in Mef2c-/- recipients (p<0.01) (**Figure 4B**). This was unexpected, given that Mef2c deletion had led to increased Treg suppressive function *in vitro* (**Figure 1**). Nevertheless, histologic comparison of grafts harvested from the 2 groups at 21 days post-transplant showed only focal interstitial infiltrates in WT recipients but dense mononuclear infiltrates with multifocal myocyte necrosis and vascular injury in conditionally deleted Mef2c-/- recipients (**Figure 4C**); such Mef2c-associated pathology is very similar to that seen previously following Mef2d deletion in Foxp3+ Treg cells (18). Using real-time qPCR, we compared intragraft gene expression at the 21 days post-transplant time-point in WT vs. Mef2c-/- recipients. The levels of CD4, CD8, CD19 and Foxp3 were comparable between groups, as was IL-2 expression, but Mef2c-/- recipients had increased intragraft expression of IFN-γ, granzyme-B and Ctla4 (**Figure 4D**). Hence our *in vivo* allograft studies are consistent with conditional deletion of Mef2c leading to impaired Treg function.

**Figure 5:**
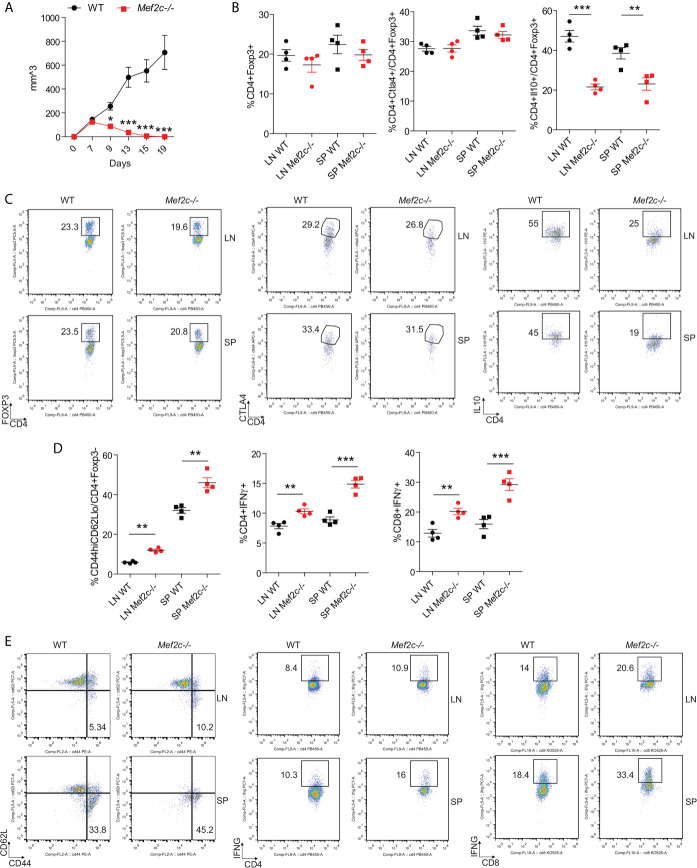
Mef2c deletion in Foxp3+ Tregs promotes anti-cancer immunity. **(A)** Graphs representing the tumor growth in 10 WT and 10 Mef2c-/- mice during a 19-day-long observation period after the subcutaneous injection of 1.2x10^6^ TC1 cells. The experiment was repeated twice with similar results (t-test between the two groups for each time point, *p < 0.05, ***p < 0.005). **(B, C)** Analysis of CD4+Foxp3+, CD4+Foxp3+Ctla4+ and CD4+Foxp3+IL-10+ populations in single-cell suspensions obtained from the draining lymph node and spleen harvested from four representative mice injected as in **Figure 5**. In the case of IL-10 production, isolated cells were stimulated for 4 h with PMA/ionomycin before the staining. (n = 4, t-test between the two groups, **p < 0.01, ***p < 0.005). **(D, E)** Analysis of the activation status and IFN-γ production in conventional CD4+ T cells and CD8+ T cells in single-cell suspensions obtained from the draining lymph node and spleen harvested from four representative mice injected as in **(A)**. (n = 4, t-test between the two groups, **p < 0.01, ***p < 0.005).

Second, we studied the effects of Mef2c deletion on anti-tumor immunity, by using syngeneic models of lung cancer (TC1 tumor cells injected subcutaneously). After the successful engraftment and an initial proliferative phase, the growth of TC1 cells was impaired in Mef2c-/- mice and the tumors were completely cleared in all of the 10 Mef2c-/- mice evaluated (**Figure 5A**, with individual growth curves shown in **Supplementary Figure 2**). Equal proportions of CD4+Foxp3+ and CD4+Foxp3+Ctla4+ cells were observed in the draining lymph nodes and spleens of WT and Mef2c-/- mice (**Figures 5B, C**), while Mef2c-/- Tregs showed impaired production of IL-10 (**Figures 5B, C**). The fast kinetics of tumor rejection did not allow us to quantify the tumor infiltration of CD4+ and CD8+ cells in Mef2c-/- mice, but we observed higher activation (CD44^high^/CD62L^low^) and greater IFN-γ production by CD4 and CD8 (**Figures 5D, E**) T cells in the tumor-draining lymph nodes and spleens of Mef2c-/- mice versus WT tumor-bearing mice. As with the allograft data (**Figure 4**), these tumor studies indicated impaired Treg function *in vivo* after conditional Mef2c deletion. Together, these data are indicative of a marked impairment of Mef2c-/- Tregs *in vivo* with characteristics and a magnitude of responses very similar to those described for the conditional deletion of Mef2d (18).

## Discussion

While our *in vitro* analysis suggested Mef2c-/- Tregs have enhanced suppressive function, our molecular, biochemical and *in vivo* data all clearly point to disruption of normal Treg function upon deletion of Mef2c. Such a disconnect has been reported by us (15) and others (41–43) and is thought to reflect the limitations of the standard *in vitro* Treg suppression assay. Indeed, the originator of the *in vitro* Treg suppression assay has reviewed this topic (44), concluding that there are dozens of examples of genes whose deletion in Tregs has differing effects *in vitro* and *in vivo*, and that there is major need for improvements to the *in vitro* assay so as to better predict likely *in vivo* effects. We conclude that Mef2c is another example of this disconnect and going forward have chosen to focus on the molecular, biochemical and *in vivo* effects of Mef2c deletion on Treg biology.

The complex and dynamic nature of the protein complexes that dictate Treg identity obey biological, biochemical and epigenetic features that similarly control many other differentiative and adaptive processes (11). Similarly to Foxp3, Mef2 TFs assemble and disassemble dynamically with co-activators and co-repressors to pursue the plastic control of the transcriptional program (19). In Treg cells, Mef2d is required to sustain and integrate the Foxp3 transcriptional repertoire (18); *Mef2d* deletion in Foxp3+ cells impairs the acquisition of an eTreg phenotype and leads to an increase in anti-cancer immunity and transplant rejection (18). Although Mef2c is expressed at very low levels in Tregs, *Mef2c-/-* mice rapidly reject heart allografts and restrain cancer growth in syngeneic models, similarly to *Mef2d-/-* mice. The de-repression of Hdac9 achieved after Mef2c KO plays a central key role in blocking Mef2d responses and Treg immune suppressive functions. Hdac9 is transcribed by Mef2d as part of a physiological feedback aimed at switching-off Mef2d transcriptional program (23, 45). This feedback mechanism is active in Tregs (18) and Hdac9-/- Tregs have increased suppressive functions (33, 46). Interestingly, the strength of this feedback mechanism is altered in some pathological conditions, such as cardiac hypertrophy (47) and cancer (23, 48). However, the genetic and epigenetic factors that regulate this feedback mechanism are not yet known.

Here we have added a new level of complexity to the well-known molecular circuit involving Mef2 TFs and their strongest repressors, the class IIa HDACs. We report the existence of an alternative mechanism through which two different paralogues of Mef2 family, Mef2c and Mef2d, respectively repress and promote Hdac9 transcription. Although we have not yet clarified the detailed mechanism by which Mef2c maintains the repression of Hdac9, a suggestive hypothesis that is corroborated by the first ChIP data is that two antagonist complexes are assembled on Hdac9 promoter: an activator complex bound to Mef2d and a repressive one complexed to Mef2c. The presence of such delicate balances underlines the central role played by Mef2 TFs in supporting Treg identity and encourages further studies to clarify the molecular details of these interactions.

## Data Availability Statement

The datasets presented in this study can be found in online repositories. The names of the repository/repositories and accession number(s) can be found below: https://www.ncbi.nlm.nih.gov/, GSE139480.

## Ethics Statement

Animal studies were approved by the Institutional Animal Care and Use Committee of the Children’s Hospital of Philadelphia (protocols 17-001047 and 19-000561).

## Author Contributions

EG designed and performed experiments, analyzed data and drafted the manuscript. LW performed cardiac allografts, performed experiments, and analyzed data. YX performed experiments and analyzed data. LC performed experiments and analyzed data. TA performed experiments and analyzed data. RH provided technical assistance. AB performed experiments. MT provided technical support. CB analyzed data. UB provided assistance with RNA-seq studies. WH analyzed data and oversaw the experimental design and writing of the manuscript. All authors contributed to the article and approved the submitted version.

## Funding

This work was supported by NIH grants R01 AI12324 and R01 CA177852 (to WH). EG received a “Fondazione Umberto Veronesi Fellowship”.

## Conflict of Interest

The authors declare that the research was conducted in the absence of any commercial or financial relationships that could be construed as a potential conflict of interest.

## Supplementary Material

Supplementary Figure 1 Treg suppression assays showing proliferation of CD4 T cell responders (upper panel) and CD8 T cell responders (low panel). These are from the same experiment performed in triplicate (Trial 2) as shown in **Figure 1F** (total T cell responders). These experiments were repeated two times by using three mice per group.

Supplementary Figure 2 Mef2c deletion promotes anti-cancer immunity. Graphs representing the detailed tumor growth data for each of the 10 WT and 10 Mef2c-/- mice during a 19-day-long observation period after the subcutaneous injection of 1.2x10^6^ TC1 cells. The experiment was repeated twice with comparable results.
