# Supplementary figures and images for: A Biological Circuit Involving Mef2c, Mef2d, and Hdac9 Controls the Immunosuppressive Functions of CD4+Foxp3+ T-Regulatory Cells
Source: Front Immunol. 2021 Jul 5;12:703632. doi: 10.3389/fimmu.2021.703632 (PMC8287581; doi:10.3389/fimmu.2021.703632)

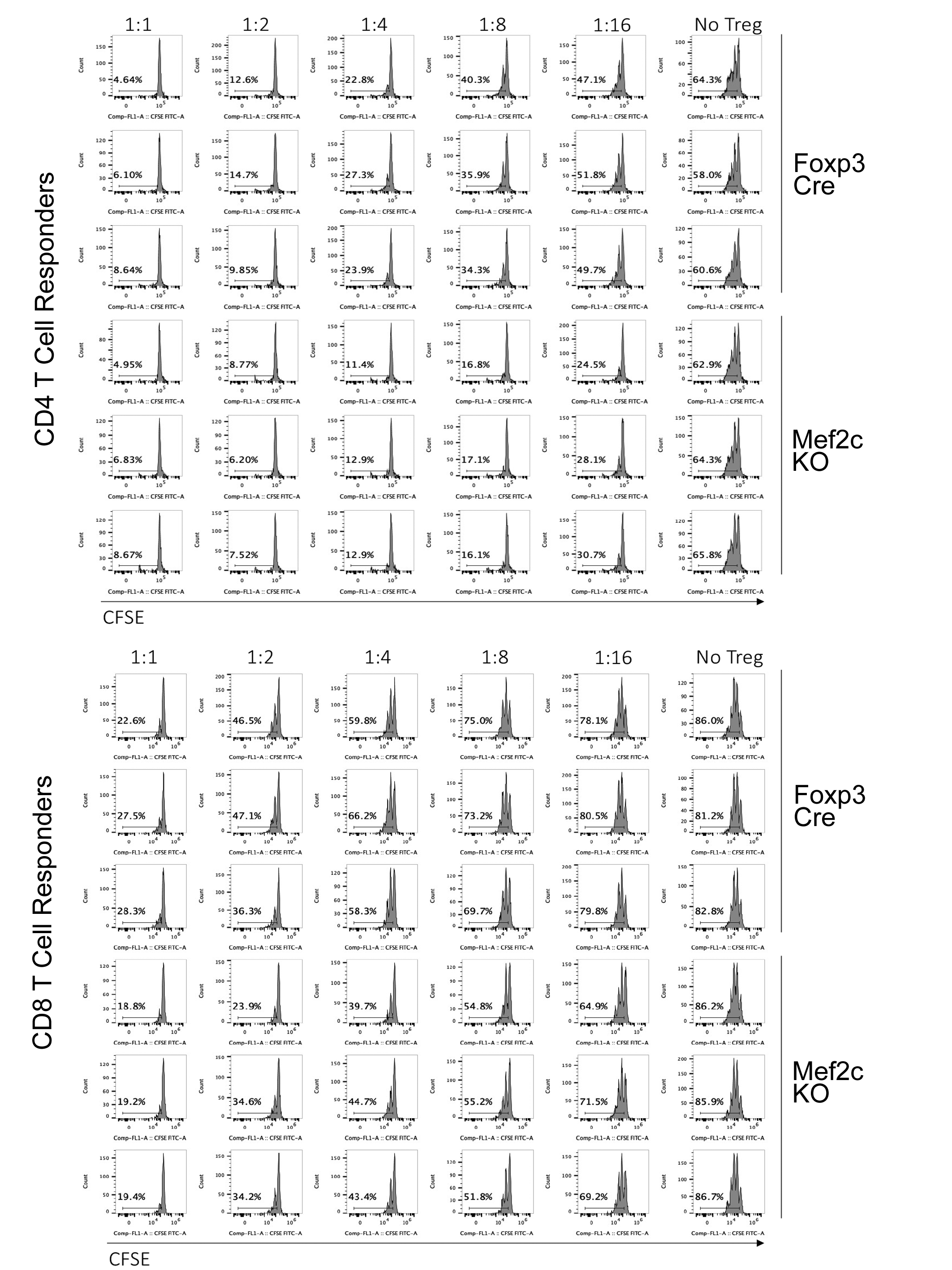

Supplement: Supplementary Figure 1 — Treg suppression assays showing proliferation of CD4 T cell responders (upper panel) and CD8 T cell responders (low panel). These are from the same experiment performed in triplicate (Trial 2) as shown in Figure 1F (total T cell responders). These experiments were repeated two times by using three mice per group. [file Image_1.tif]

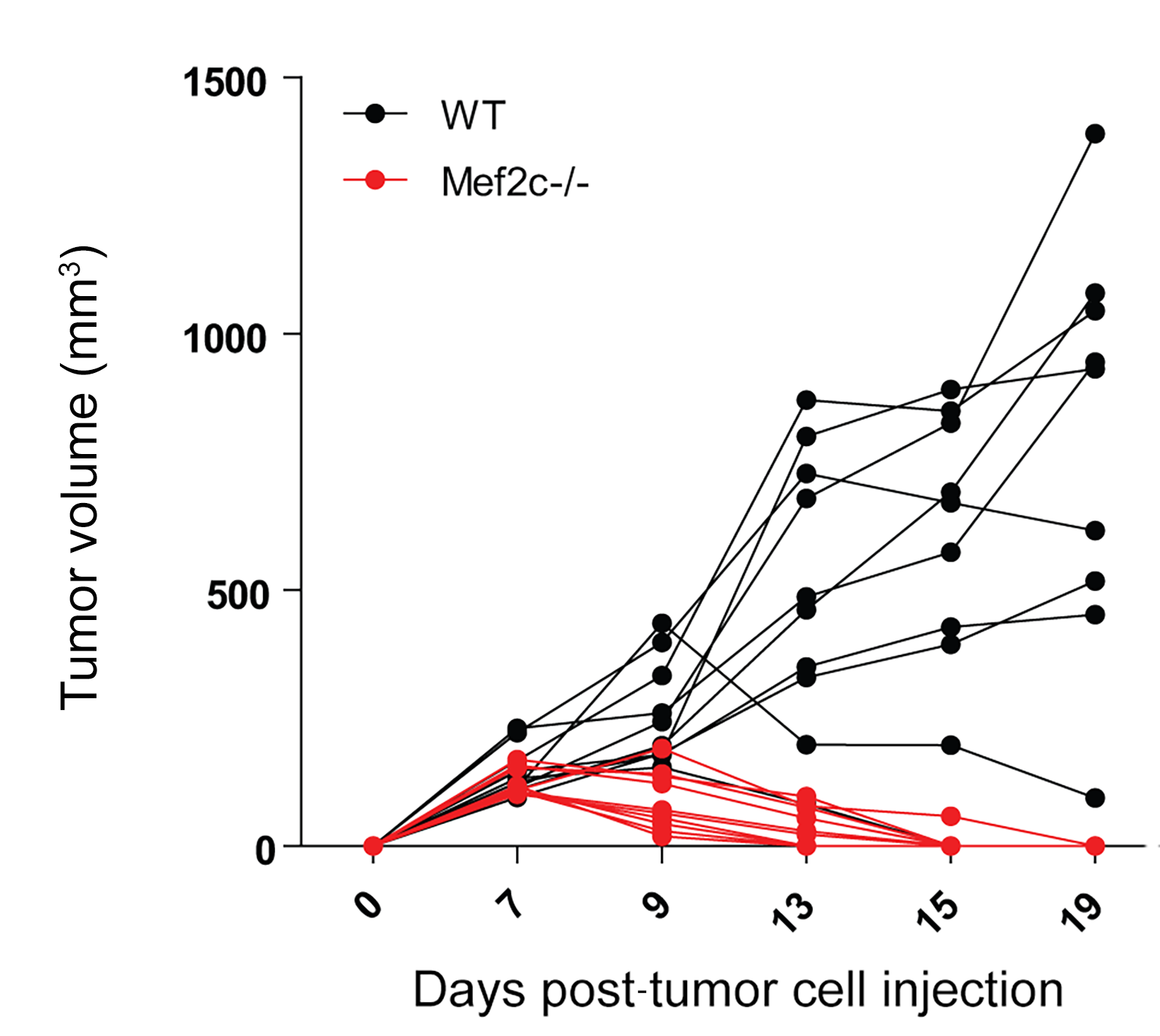

Supplement: Supplementary Figure 2 — Mef2c deletion promotes anti-cancer immunity. Graphs representing the detailed tumor growth data for each of the 10 WT and 10 Mef2c-/- mice during a 19-day-long observation period after the subcutaneous injection of 1.2x10^6^ TC1 cells. The experiment was repeated twice with comparable results. [file Image_2.tif]
